# Inhaled corticosteroid treatment for 6 months was not sufficient to normalize phagocytosis in asthmatic children

**DOI:** 10.1186/2045-7022-3-28

**Published:** 2013-08-30

**Authors:** Carmen Lívia Faria da Silva-Martins, Shirley Claudino Couto, Maria Imaculada Muniz-Junqueira

**Affiliations:** 1 Laboratory of Cellular Immunology, Pathology, Faculty of Medicine, Campus Darcy Ribeiro, Asa Norte, University of Brasilia, Brasilia, DF 70.910-900, Brazil; 2 Department of Paediatric, Faculty of Medicine, Campus Darcy Ribeiro, Asa Norte, University of Brasilia, Brasilia, DF 70.910-900, Brazil; 3 Paediatric Service, University Hospital of Brasilia, Brasilia, DF 70.910-900, Brazil

**Keywords:** Asthma, Immunodeficiency, Neutrophils, Monocytes, Oxygen radical production, Phagocytosis

## Abstract

**Background:**

Corticosteroids are the first-line therapy for asthma; however, the effect of corticosteroids on the innate immune system remains unclear. This study’s objective was to evaluate the effect of inhaled corticosteroid therapy (ICT) on phagocytic functions.

**Methods:**

To evaluate the impact of ICT, the phagocytosis of *Saccharomyces cerevisiae* by blood monocytes and neutrophils and the production of superoxide anions were assessed before and after three and six months of ICT treatment in 58 children with persistent asthma and 21 healthy controls.

**Results:**

We showed that the phagocytic capacity of monocytes and neutrophils that occurred via pattern recognition receptors or was mediated by complement and immunoglobulin receptors in asthmatic children before treatment was significantly lower than in healthy controls (p<0.05, Mann–Whitney test) and was not influenced by the severity of the clinical form of the disease. Although there was clinical improvement with treatment, ICT for 6 months was not sufficient to normalize phagocytosis by the phagocytes. Superoxide anion production was also decreased in the asthmatic children before treatment, and ICT normalized the O^-^ production only for children with mild persistent asthma when assessed at baseline but caused this function to decrease after stimulation (p<0.05, Kruskal-Wallis test).

**Conclusions:**

Our data suggest that an immunodeficiency in phagocytes remained even after treatment. However, this immunodeficiency does not appear to correspond with the clinical evolution of asthma because an improvement in clinical parameters occurred.

## Introduction

Asthma is a serious global health problem throughout the world. An estimated 300 million individuals are affected by this disease [1], which is the most common chronic disease of childhood [2].

Corticosteroids are the first-line drugs for asthma therapy and are by far the most effective anti-inflammatory treatment [3], resulting in marked reductions in asthma morbidity and mortality [4,5].

The goals of asthma treatment are to control the clinical symptoms for extended periods, prevent asthma exacerbations, maintain pulmonary function, and control airway inflammation [6]. Inhaled corticosteroids (ICS) are very effective in controlling asthma symptoms in patients of all ages and severities and may prevent irreversible airway changes [4,5,7]. However, corticosteroid treatment also has several side effects [8] and may influence the functions of immune system cells [9,10].

Children with asthma have an increased frequency of pulmonary infections, and it is possible that alterations in the function of phagocytes, which are engaged in the first line of defense against pathogens, may play a role in these infections [11]. Monocyte chemotaxis *in vitro* is inhibited by high concentrations of steroids [12]. In addition, corticosteroids affect nitric oxide production, total free radical production, and nitric oxide synthase activity in the monocytes of asthmatic patients [10]. Furthermore, there are reports of defective phagocytosis of pathogens in asthma patients [13]. Phagocytosis by alveolar macrophages is impaired in children with poorly controlled asthma [11], and oxidative stress is emerging as a common mechanism that may alter both macrophage and neutrophil functions [13].

The influence of ICS treatment on phagocyte functions in asthmatic children remains unclear. Either the treatment or the disease may influence phagocyte functions directly or through several cytokines and substances produced as part of the altered immune response in affected individuals. Therefore, the objective of this study was to evaluate the undetermined influence of ICS treatment on the phagocytic function of monocytes and neutrophils from asthmatic children after 3 and 6 months of therapy and to compare phagocytosis and the production of toxic oxygen radicals in asthmatic children and healthy control subjects. This study may shed light on the effects of corticosteroids on cells of the innate immune system in asthmatic children and may improve the understanding of the mechanism of action of this drug. It was showed that the phagocytic capacity of monocytes and neutrophils that occurred via pattern recognition receptors or was mediated by complement and immunoglobulin receptors in asthmatic children before treatment was significantly lower than in healthy controls and inhaled corticosteroid treatment for 6 months was not sufficient to normalize phagocytosis in asthmatic children.

## Methods

### Study groups

One author (CLFSM), a pediatric pneumologist, conducted the clinical evaluation of all control and asthmatic children and sequentially selected and screened 58 children who were seen in the pediatric asthma outpatient clinic at Brasilia University Hospital for enrolment in this study. The children were placed in the following groups: a group of 58 outpatient asthmatic children with variable disease severity (27 girls and 31 boys; 7.6 ± 3.4 years) from whom blood was collected before, at 3 months, and at 6 months of inhaled corticosteroid therapy. Asthma was classified as mild, moderate, or severe persistent disease by the frequency of the symptoms, presence of nocturnal asthma, frequency of acute exacerbations, medications required for control, physical activity limitations, or altered pulmonary functioning [14]. Twenty-four (41.3%) asthmatic children had mild asthma, 20 (34.5%) children had moderate asthma and 14 (24.1%) had severe persistent asthma. A group of 21 healthy children (12 girls and 9 boys; 10.7 ± 2.10 years) without the disease and without a personal or familial history of allergy comprised the control group. At the time of clinical examination, peripheral blood was collected to assess phagocytosis and superoxide anion production.

The exclusion criteria were as follows: children < 2 years or > 18 years, any other clinically significant pulmonary disease or other disease, those who presented with any condition that might alter the function of the immune system, any children taking medication other than asthma treatment, and any children that had previously used inhaled corticosteroids, or that used oral corticosteroids in the last 6 months.

During the first evaluation, the group of asthmatic children was assessed for the severity of asthma, asthma control test (ACT), peak expiratory flow (PEF), and body mass index (BMI). The ACT was administered to children aged 4–11 years (7 answers) and >12 years (5 answers). The test was adapted for children < 4 years, with the mother responding to the questions. The children were considered clinically controlled (ACT ≥ 25), partially controlled (ACT = 20–24) or uncontrolled (ACT < 20) [15].

The PEF value was recorded as the best of three forced expirations by means of a peak flow meter and expressed as the percentage of predicted of normal values for height and sex values for children ≥ 100 cm. In seven children with mild persistent asthma (mPA), one child with moderate persistent asthma (MPA) and three with severe persistent asthma (SPA) the PEF were not assessed. All these children younger than 5 years had familial history of asthma. The thresholds of PEF used to severity evaluation were: mPA ≥80% predicted value, MPA= 60-80% predicted value, and SPA ≤ 60% predicted value.

BMI was assessed to exclude any possible influence of child nutritional status on phagocyte functions.

During the second (3 months) and third (6 months) clinical follow-up visits, the children were assessed with the ACT, PFE and BMI.

The children were treated with ICS. A long-acting β_2_-agonist (LABA) was added when necessary to control symptoms. All 24 children with mild persistent asthma (mPA), 10 children <4 years with moderate persistent asthma (MPA) and 5 children < 4 years with severe persistent asthma (SPA) received monotherapy with 250 μg beclomethasone dipropionate twice day. Eight children with MPA and three with SPA received budesonide + formoterol 6/200 μg twice day and 1 children with SPA received 12/400 μg twice day. Two children with MPA and five children with SPA received fluticasone propionate + salmeterol 25/125 μg twice day (Table 1).

**Table 1:** Clinical characteristics of healthy controls and asthmatic children before inhaled corticosteroid therapy showing mild, moderate and severe persistent asthma

**Parameters**	**Normal control**	**Mild persistent asthma**	**Moderate persistent asthma**	**Severe persistent asthma**	**Test p**
Number	21/78	24/58 (41.3%)	20/58 (34.5%)	14/58 (24.1%)	
Gender (boys/girls)	9/12	13/11	12/8	6/8	Chi-square p=0.64
Age (years) (mean±SD)	10.7±2.1	6.32±2.99	9.22±3.33	7.24±3.53	ANOVA* p<0.05
Total leukocytes	7390	9720	8120	8305	Kruskal-Wallis p=0.05
Neutrophils	3440	4150	3640	4260	Kruskal-Wallis p=0.66
Monocytes	568	568	536	643	Kruskal-Wallis p=0.57
Eosinophils	245	460	635	487	ANOVA p=0.003
BMI (Kg/m^2^) (mean±SD)	43.31±32.1	51.9±34.2	47.4±31.2	63.7±32.5	Kruskal-Wallis p=0.29
PEF (%) (mean±SD)	86.9±18.6	81.2±17.3	75.6±15.3	65.6±12.0	ANOVA** p<0.05
Treatment***					
ICS (%)		23/23 (100%)	10/20 (50%)	05/14 (35.7%)	
ICS + LABA (%)					
Budesonide + formoterol		0/23 (0%)	08/20 (40%)	04/14 (28.6%)	
Fluticasone propionate + salmeterol		0/23 (0%)	02/20 (10%)	05/14 (35.7%)	

*BMI* Body Mass Index (percentile), *PEF* Peak Expiratory Flow, *mPA* mild persistent asthma, *MPA* moderate persistent asthma, *SPA* severe persistent asthma, *mPA, MPA, SPA* data before treatment. Beclomethasone dipropionate = 250 μg to 500 μg/day; Budesonide + formoterol 6/200 μg or 12/400 μg twice day; Fluticasone propionate + salmeterol = 25/125 μg to 50/250 μg twice day K-W= Kruskal-Wallis + Dunn’s method; * mPA and SPA < C; ** SPA < C; *** Treatment followed GINA.

Peripheral blood was collected from control and asthmatic children who had fasted for > 12 hours prior to their blood being drawn. Hemogram (Cell-dyn 3.700) was automatically assessed.

The Human Research Ethical Committee of the School of Medicine of the University of Brasilia approved the experimental protocol (process no. 02/2007).

All parents provided formal informed consent for their child’s participation in this study.

### Phagocytosis test

Phagocytosis of *Saccharomyces cerevisiae* was adapted from a previously described technique [16]. Briefly, 40 μL samples per marked area of heparinized whole peripheral blood obtained from each subject were placed on duplicate slides containing 8 areas that were each 7-mm in diameter and incubated in a humidified chamber for 45 min at 37°C. The slides were then rinsed with 0.15 M phosphate-buffered saline (PBS) (pH 7.2) at 37°C to remove non-adherent cells. Adherent cells (12,534±5,050 cells/marked area; 5.63±0.85% monocytes and 93.5±1.08% neutrophils) (viability > 98%) were incubated with a suspension of 2.5×10^5^*S. cerevisiae* in 20 μL Hanks-Tris solution (Sigma Co., St Louis, MO, USA) (pH 7.2) containing 10% heat-inactivated fetal calf serum (FCS) (Gibco/Invitrogen, Grand Island, NY, USA) for 30 min in a humidified chamber at 37°C. The slides were then rinsed with 0.15 M PBS at 37°C to eliminate non-phagocytosed *S. cerevisiae*, and the final wash step was performed with 30% FCS in Hanks-Tris solution. The slides were fixed with methanol and stained with 10% Giemsa stain. The number of *S. cerevisiae* that were phagocytosed by either 200 monocytes or 200 neutrophils in individual preparations was assessed by light microscopy. The phagocytic index was calculated as the mean number of phagocytosed *S. cerevisiae* per phagocytosing monocyte or neutrophil multiplied by the percentage of these cells engaged in phagocytosis.

The internalization of particles by phagocytes occurs via receptors. When phagocytosis occurs via pattern-recognition receptors, the phagocyte recognizes directly conserved pattern molecular in the surface of the particle to be phagocytosed. When phagocytosis is facilitated by opsonins, the ingestion occurs via receptors to components of complement or via receptors to FcIgG. *Saccharomices cerevisiae* (Baker’s yeasts) suspensions were prepared according to a previously described technique [16] to assess phagocytosis via pattern-recognition receptors and facilitated by opsonins. Yeasts were used with or without previous incubation with fresh serum from the donor. In the former case, yeast cells were considered sensitized, because they were opsonized by complement molecules and antibodies in serum. These molecules adhere on yeast surface and will be recognized by their neutrophil and monocyte receptors (CR1, CR3 and FcR) during the process respective of phagocytosis [16]. Yeast cells that were not pre-incubated with fresh serum from the donor, but were incubated with fetal calf serum, were considered as non-sensitized, because they were non-opsonized and their phagocytosis occurs via the pattern-recognition receptors (PRRs) of neutrophils and monocyte [17]. For opsonization, the *S. cerevisiae* were sensitized by incubation at 37°C for 30 min with 10% fresh serum from the donor in Hanks-Tris solution. The yeast cells that were pre-incubated with inactivated fetal calf serum were considered non-sensitized, and their phagocytosis occurred via the pattern-recognition receptors of phagocytes [17].

### Nitro blue tetrazolium slide test

The nitro blue tetrazolium (NBT) test evaluated the ability to generate toxic oxygen radicals (superoxide anion/O_2_^−^) that are capable of reducing the compound NBT to an insoluble form, formazan, which is identified via optical microscopy as a blue color in the cytoplasm of the cell [18]. The amount of reduced NBT is directly proportional to the amount of oxygen radicals (O_2_^−^) produced by the phagocytes. After the phagocytes were adhered, cells from the control and asthmatic children were incubated with 0.05% NBT solution in Hanks-Tris solution (Sigma, St Louis, MO, USA) for 20 min at 37°C in a humidified chamber. The slides were then washed, fixed with methanol and stained with a 1.4% safranin and 28.6% glycerol. The percentage of phagocytes that contained reduced cytoplasmic NBT was assessed by optical microscopy, and the source of the individual preparations was revealed only at the end of the evaluation [18].

### Statistical analysis

The data were previously tested with Bartlett’s test for equal variances and the Kolmogorov–Smirnov test for normality of distribution. The Mann–Whitney test was used to compare two non-normal samples. The Kruskal-Wallis test, followed by Dunn’s method, was used to compare multiple non-normal samples. Spearman’s test was used to evaluate the correlation between samples. The chi-square test was used to compare proportions. For homogeneity of data presentation, all values were expressed as the median, quartiles and extremes, and outlier values were indicated. Differences and correlations with a two-tailed value of p<0.05 were considered statistically significant. The Prism 5 software package (GraphPad, San Diego, CA, USA) was used for statistical tests and graphical presentation of the data.

## Results

### Clinical and demographic characteristics

The characteristics of the children enrolled in the study and the clinical outcomes during 3 and 6 months of corticosteroid treatment are summarized in Tables 1 and 2.

**Table 2:** Clinical characteristics of asthmatic children before, 3 and 6 months after inhaled corticosteroid therapy

**Parameters**	**Before**	**3 months**	**6 months**	**Test p**
PEF (%) (mean±SD)				ANOVA
mPA	81.2±17.3	90.6±23.6	99.2±18.4	p < 0.05*
MPA	75.6±15.3	79.3±16.8	86.3±18.3	p < 0.05*
SPA	65.6±12.0	72.8±17.0	77.9±19.8	p > 0.05
BMI (Kg/m^2^) (mean±SD)				
mPA	51.9±34.3	45.2±32.2	43.4±37.5	Kruskal-Wallis p>0.05
MPA	47.4±31.2	50.5±31.5	50.0±30.5	
SPA	63.7±32.5	61.8±29.6	63.2±29.5	
ACT n (%)				
mPA				
≥25	0/23 (0%)	1/23 (4.3%)	4/22 (18.2%)	Chi-square
20-24**	11/23 (47.8%)	19/23 (82.6%)	16/22 (72.7%)	p<0.05
<20***	12/23 (52.2%)	3/23 (13.0%)	2/22 (9.1%)	
MPA				
≥25	0/20 0%)	1/20 (5%)	3/20 (15%)	Chi-square
20-24**	2/20 (10%)	7/20 (35%)	10/20 (50.0%)	p<0.05
<20***	18/20 (90%)	12/20 (60%)	7/20 (35.0%)	
SPA				
≥25	0/14 (0%)	0/14 (0%)	0/14 (0%)	Chi-square
20-24	2/14 (14.3%)	3/14 (21.4%)	4/14 (28.6%)	p>0.05
<20	12/14 (85.7%)	11/14 (78.57%)	10/14 (71.4%)	

*PEF* Peak Expiratory Flow, *BMI* Body Mass Index (percentile), *ACT* Asthma Control Test, *mPA* mild persistent asthma, *MPA* moderate persistent asthma, *SPA* severe persistent asthma, * 6 m > before ** 3 m and 6 m > before *** 3 m and 6 m < before.

Children from the control group had the mean age 3 years older than the asthmatic children (10.7 ± 2.10 years *versus* 7.6 ± 3.4 years; p=0.001, t test), but no difference was observed based on gender between the children with or without asthma (p=0.6, Chi-square test). To exclude a possible influence of age as an independent confounding variable on the results of phagocyte functions, the univariate correlation between age and monocyte and neutrophil phagocytic indices and % NBT reduction for healthy control and asthmatic children was tested. No correlation was observed between age and these phagocyte functions (Spearman’s test, p>0.05). Therefore, no adjustment of results of phagocyte functions for age was done. Furthermore, previous observations had shown that there are not differences among preschool children, schoolchildren and adolescent phagocyte functions.

No statistically significant difference was observed between groups for leukocytes, except for eosinophils. The median of the number of eosinophils in peripheral blood was significantly higher in asthmatic children than healthy control (245 for the control group and 460, 635, and 487 for mPA, MPA and SPA groups, respectively, before ICT; p=0.003, ANOVA) (Table 1).

ACT and PEF showed the clinical improve after 3 and 6 months follow up (Tables 1 and 2).

In the 3 months evaluation, the phagocyte tests was done in 50 children (n = 50), and in the 6 months evaluation, the phagocyte tests of was done in 47 children (n = 47).

### No difference was observed between the phagocytic indices in children with different clinical severities of persistent asthma

**Figure 1:**
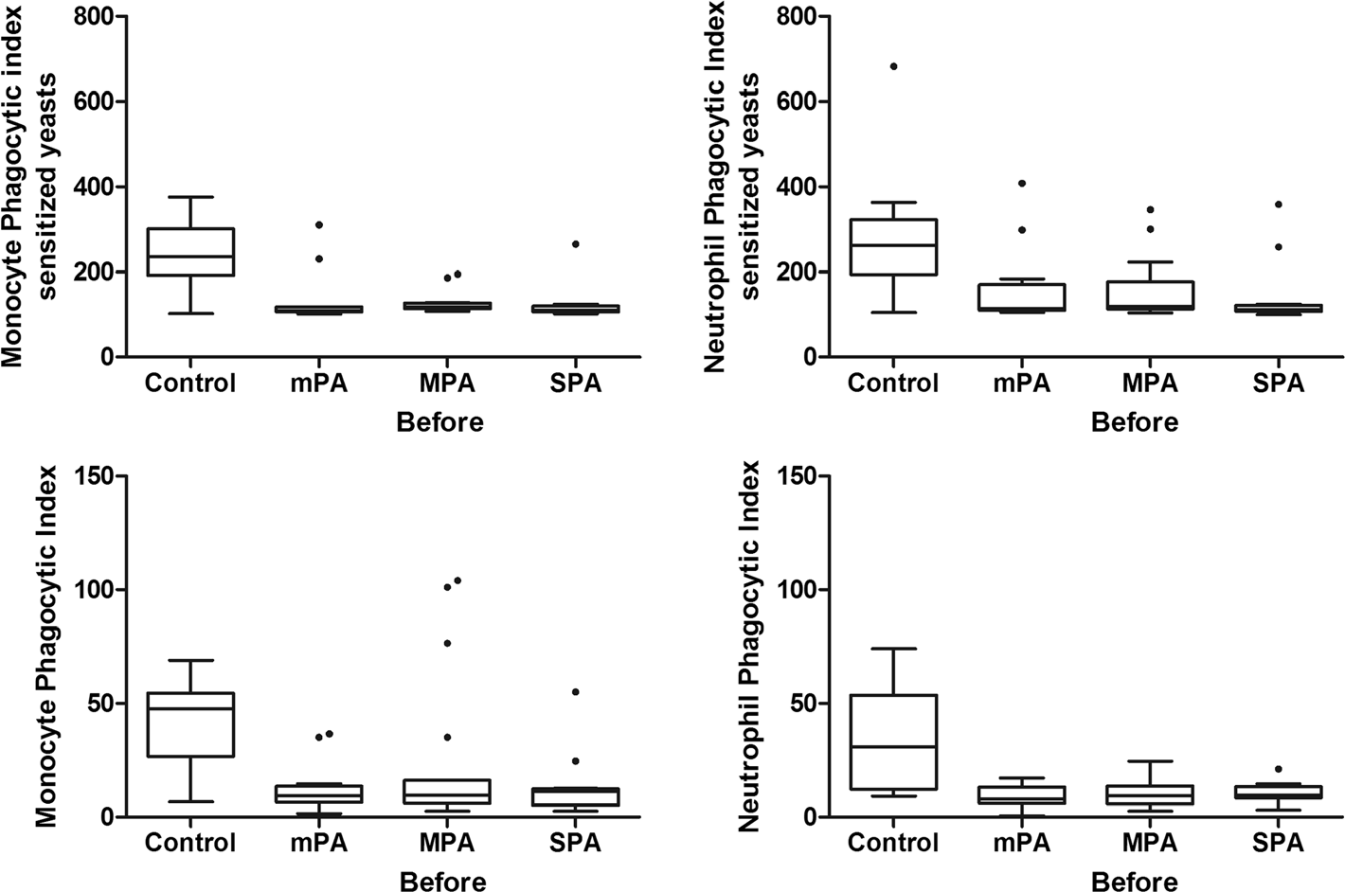
***In vitro *evaluation of the phagocytic indices of monocytes (left) and neutrophils (right) in individuals with mild persistent asthma (mPA), moderate persistent asthma (MPA) and severe persistent asthma (SPA) before treatment and in normal control children using 2.5×10**^**5 **^**yeast cells per well.** For the top panels, sensitized yeasts were used. For the bottom panels, non-sensitized yeasts were used. Statistical analyses showed that the median value of all groups of asthmatic children was lower than that of the healthy control children; however, no difference was observed between the groups with mild, moderate and severe persistent asthma (Kruskal-Wallis test followed by the Dunn’s method). The data are expressed as the median, quartile and extreme, and outlier values are marked.

To determine whether the severity of persistent asthma influenced the phagocytic capacity of monocytes and neutrophils, the phagocytic indices of monocytes and neutrophils from children with different severities of asthma were compared. All groups of asthmatic children showed a lower phagocytic index than the healthy control children; however, no difference was observed between the children with mild, moderate or severe persistent asthma (Kruskal-Wallis test followed by Dunn’s method, p<0.01) (Figure 1).

### Monocytes and neutrophils from children with asthma showed less phagocytosis through pattern molecular receptors, and treatment with ICS for 6 months was not sufficient to normalize phagocytic function

To assess the influence of inhaled corticosteroids on phagocytosis, the phagocytic capacity of monocytes and neutrophils from children with different severities of asthma was compared before and after corticosteroid treatment.

**Figure 2:**
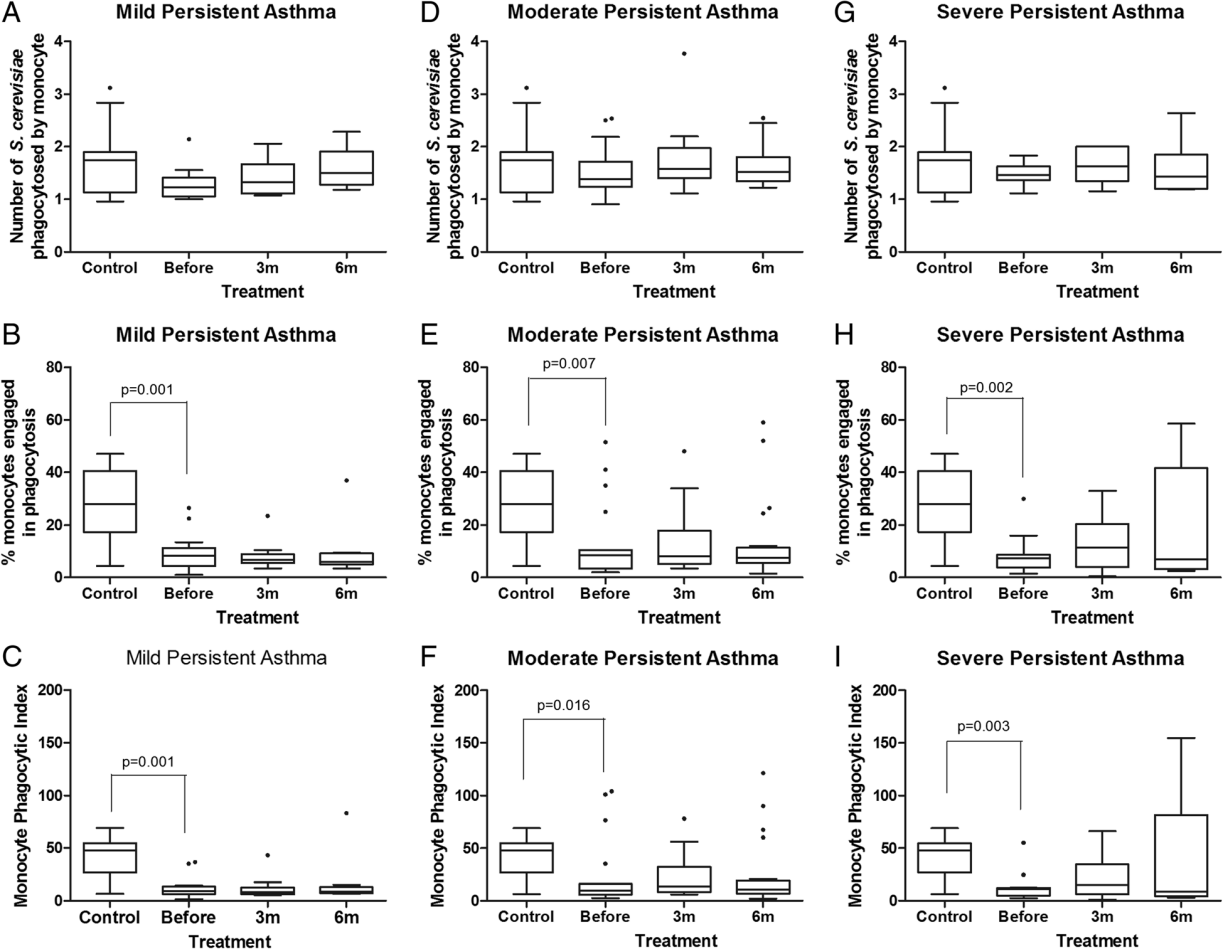
**Influence of inhaled corticosteroid treatment on the phagocytic capacity of monocytes via pattern-recognition receptors in children with mild, moderate and severe persistent asthma before and during the 3**^**rd **^**and 6**^**th **^**months of treatment compared to healthy controls using non-sensitized *Saccharomyces cerevisiae*.** The data are expressed as the median, quartile and extreme values. The outlier data are indicated. Top: average number of *S. cerevisiae* yeast cells ingested by phagocytosing monocytes **(A, D, G)**; Middle: percentage of monocytes engaged in phagocytosis **(B, E, H)**; Bottom: monocyte phagocytic index **(C, F, I)**. The statistical differences are marked in the graphics and in text.

Using non-sensitized *S. cerevisiae*, the monocytes of children with mild, moderate and severe persistent asthma showed a lower phagocytic index than healthy children. The median phagocytic index of the monocytes from children with asthma from all groups prior to treatment was significantly lower than that in the control healthy children (p<0.05, Mann–Whitney test) (Figure 2C, F, I), and this deficiency was caused by lower quantitative involvement of phagocytes in phagocytosis when compared to the control healthy children (p<0.05, Mann–Whitney test) (Figure 2B, E, H), because there was no difference in the median number of phagocytosed *S. cerevisiae* per monocyte between the control and asthmatic children (p>0.05, Mann–Whitney test) (Figure 2A, D, G). Subsequent to the inhaled corticosteroid treatment of children with asthma, there were no differences among the groups for phagocytic index, percentage of monocytes engaged in phagocytosis and the number of yeast cells phagocytosed over six months of treatment (Figure 2).

**Figure 3:**
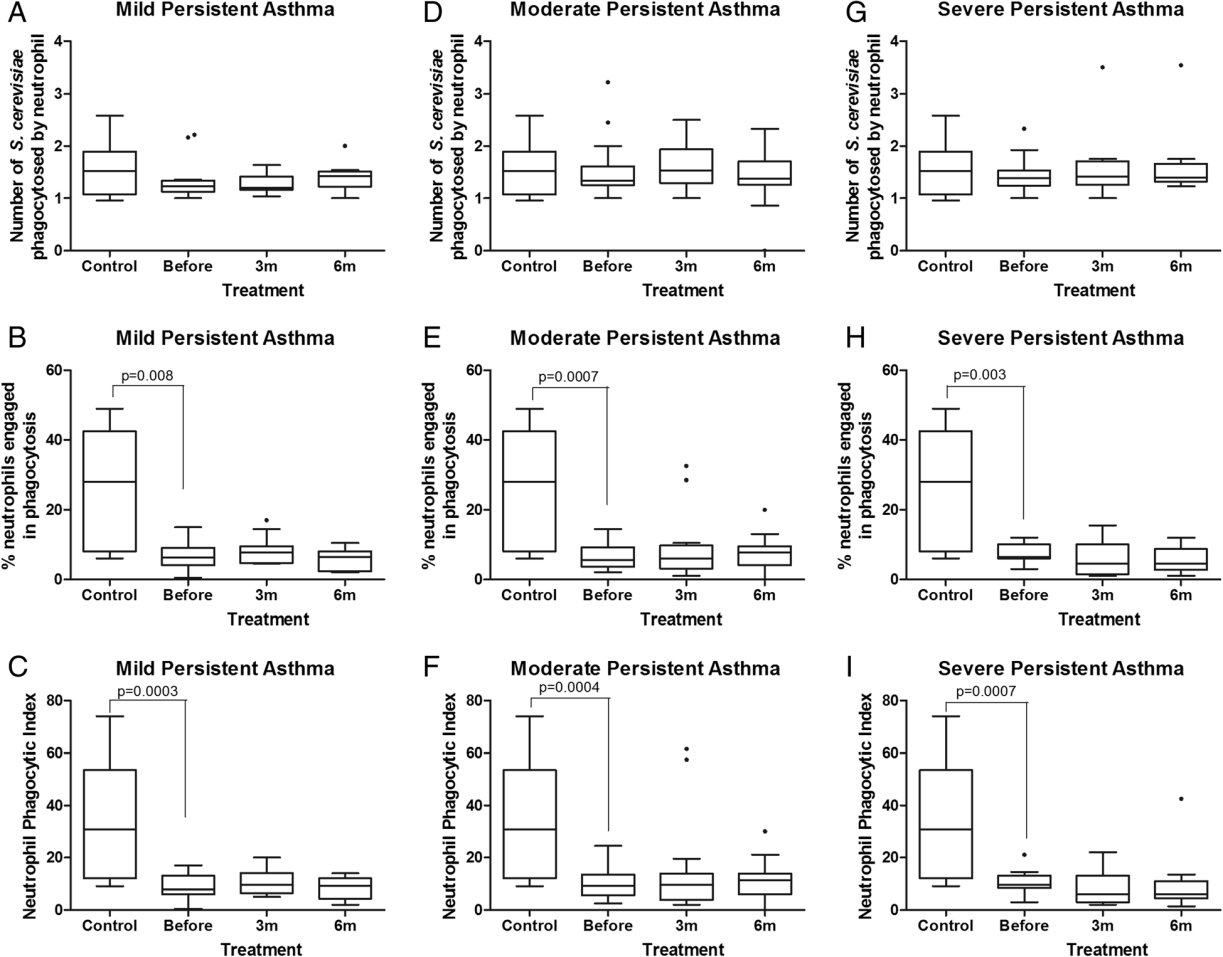
**Influence of inhaled corticosteroid treatment on the phagocytic capacity of neutrophils via pattern-recognition receptors in children with mild, moderate and severe persistent asthma before and during the 3**^**rd **^**and 6**^**th **^**months of treatment compared to healthy controls using non-sensitized *Saccharomyces cerevisiae*.** The data are expressed as the median, quartile and extreme values. The outlier data are indicated. Top: average number of *S. cerevisiae* yeast cells ingested by phagocytosing neutrophils **(A, D, G)**; Middle: percentage of neutrophils engaged in phagocytosis **(B, E, H)**; Bottom: neutrophil phagocytic index **(C, F, I)**. The statistical differences are marked in the graphics and in text.

Similar results were observed for the neutrophils. Prior to the inhaled corticosteroid treatment, the neutrophils from children with mild, moderate and severe asthma also exhibited significantly lower phagocytic indices (p < 0.05, Mann–Whitney test; Figure 3C, F, I) and lower proportions of cells involved in phagocytosis (p < 0.05, Mann-Whiney test; Figure 3B, E, H) than the neutrophils from control healthy children for non-sensitized yeast cells (Figure 3). ICS therapy for 3 and 6 months did not modify phagocytosis by the neutrophils (Figure 3).

### Monocytes and neutrophils from children with asthma showed less phagocytosis of opsonized yeast cells via complement and immunoglobulin receptors, and treatment with inhaled corticosteroids (ICS) for 6 months was not sufficient to normalize phagocyte function

**Figure 4:**
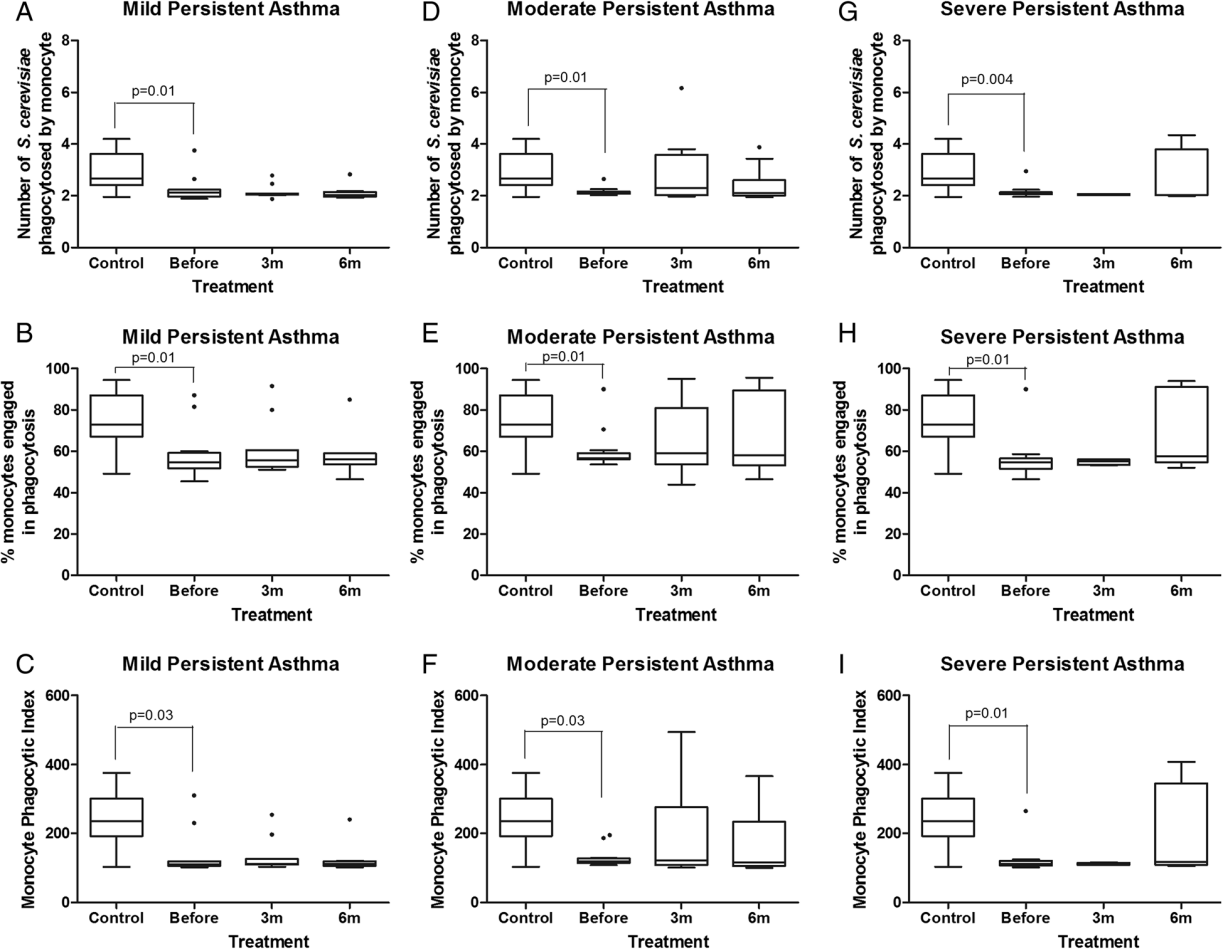
**Influence of inhaled corticosteroid treatment on the phagocytic capacity of monocytes via opsonin receptors in children with mild, moderate and severe persistent asthma before and during the 3**^**rd **^**and 6**^**th **^**months of treatment compared to healthy controls using sensitized *Saccharomyces cerevisiae*.** The data are expressed as the median, quartile and extreme values. The outlier data are indicated. Top: average number of *S. cerevisiae* yeast cells ingested by phagocytosing monocytes **(A, D, G)**; Middle: percentage of monocytes engaged in phagocytosis **(B, E, H)**; Bottom: monocyte phagocytic index **(C, F, I)**. The statistical differences are marked in the graphics and in text.

**Figure 5:**
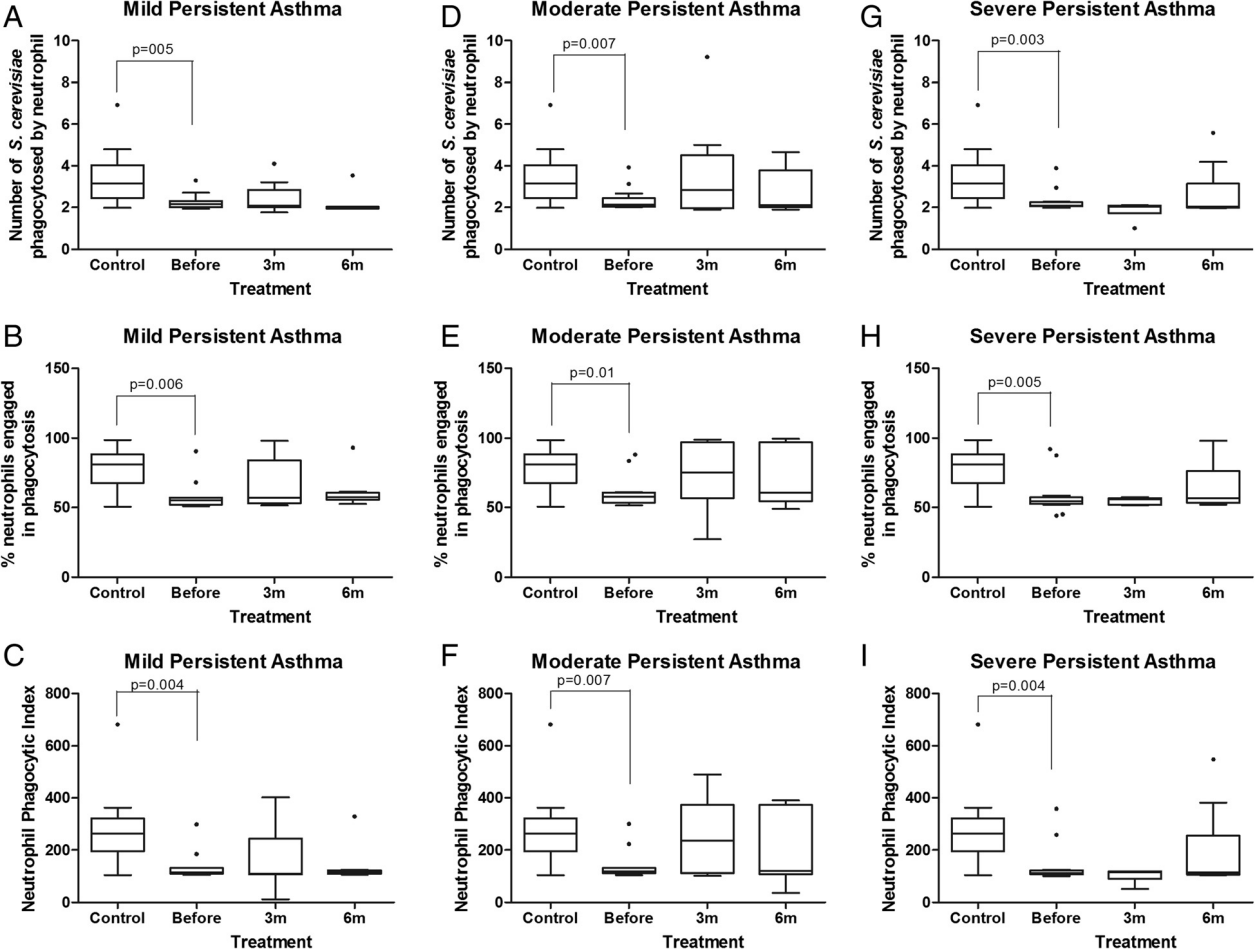
**Influence of inhaled corticosteroid treatment on the phagocytic capacity of neutrophils via opsonin receptors in children with mild, moderate and severe persistent asthma before and during the 3**^**rd **^**and 6**^**th **^**months of treatment compared to healthy controls using sensitized *Saccharomyces cerevisiae*.** The data are expressed as the median, quartile and extreme values. The outlier data are indicated. Top: average number of *S. cerevisiae* yeast cells ingested by phagocytosing neutrophils **(A, D, G)**; Middle: percentage of neutrophils engaged in phagocytosis **(B, E, H)**; Bottom: neutrophil phagocytic index **(C, F, I)**. The statistical differences are marked in the graphics and in text.

Using sensitized *S. cerevisiae*, the monocytes and neutrophils from children with mild, moderate and severe persistent asthma also showed lower phagocytic indices than the healthy children. The median phagocytic indices of monocytes and neutrophils from children with asthma from all groups prior to treatment was significantly lower than the healthy control children (p<0.05, Mann–Whitney test; Figure 4C, F, I). This result differed from that obtained using the non-sensitized yeasts. The decrease in monocyte and neutrophil phagocytic index was caused by both the lower quantitative involvement of the phagocytes in phagocytosis when compared to the healthy control children (p<0.05, Mann–Whitney test; Figure 4B, E, H; Figure 5B, E, H), and the smaller number of particles phagocytosed per monocyte or neutrophil (p > 0.05, Mann–Whitney test, Figure 4A, D, G; Figure 5A, D, G). Moreover, ICS therapy for 6 months was not able to modify the phagocytic index, the percentage of monocytes or neutrophils engaged in phagocytosis or the number of phagocytosed yeast (Figures 4 and 5).

### Influence of inhaled corticosteroids on superoxide anion production

**Figure 6:**
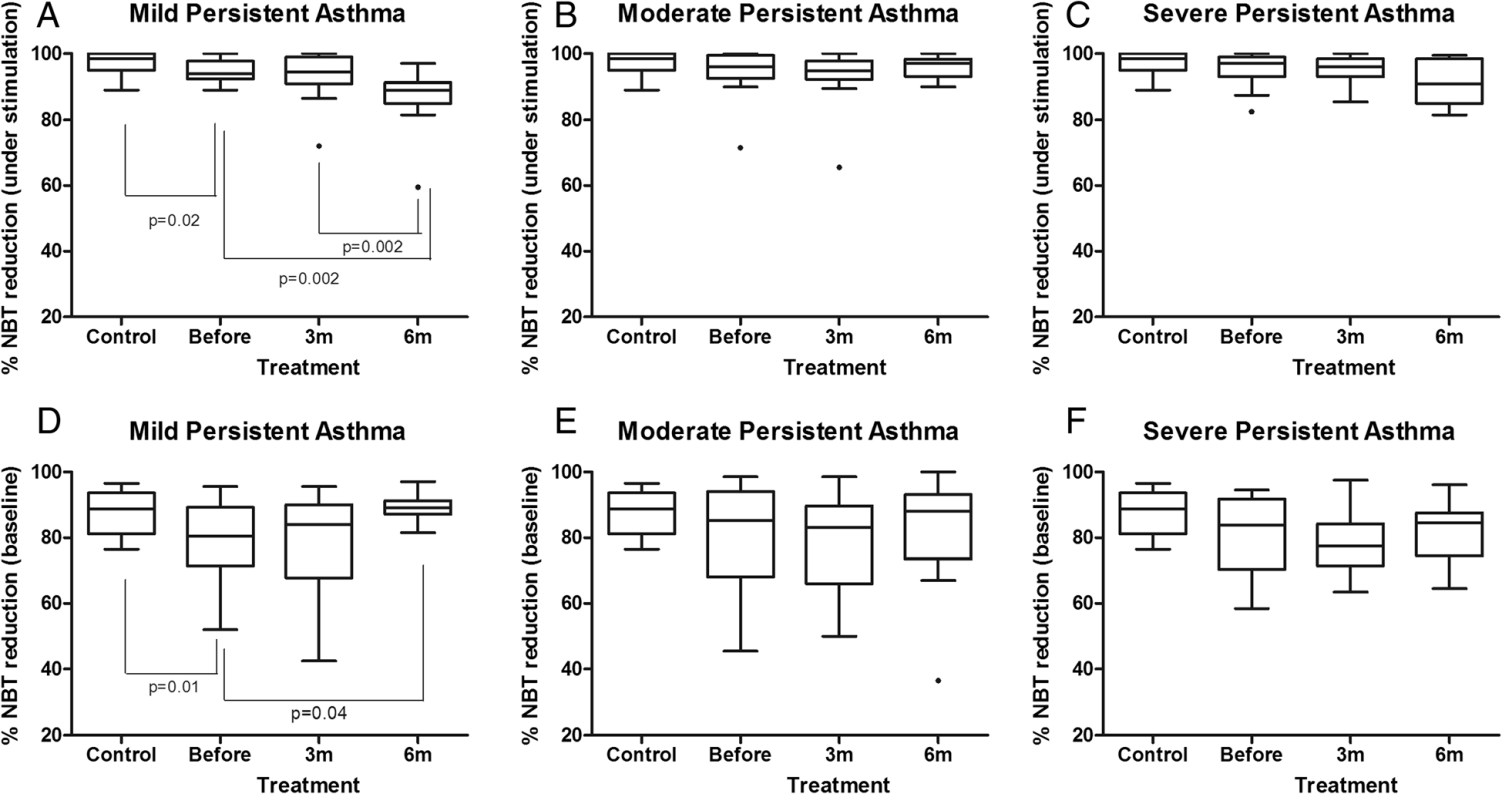
**Comparison of phagocyte production of toxic oxygen radical molecules assessed as the per cent reduction of nitro blue tetrazolium among the groups with mild, moderate and severe persistent asthma and healthy control children.** In **A, B, C**: under stimulation. In **D, E, F**: baseline. The data are expressed as the median, quartile and extreme values. The outlier data are indicated. The statistical differences are marked in the graphics and in text.

The difference between the control and asthmatic children was statistically significant only for the children with mild persistent asthma (Figure 6A, D). When the % reduction of NBT was assessed either with (p<0.02, Mann–Whitney test, Figure 6A) or without stimulation (p=0.01, Mann–Whitney test, Figure 6D), the capacity to produce superoxide anions was decreased in the asthmatic children before treatment. Treatment with inhaled corticosteroids differentially influenced the production of this oxygen radical. When assessed without stimuli, there was an increase in the % reduction of NBT after 6 m of treatment (p<0.05, Kruskal-Wallis test followed by Dunn’s method, Figure 6D). However, when the % NBT reduction was assessed after phagocytosis stimulation, there was decreased production of superoxide anions after 3 and 6 m of follow-up (p<0.05, Kruskal-Wallis test followed by Dunn’s method) (Figure 6A).

## Discussion

This prospective study evaluated for the first time the influence of 3 and 6 months of treatment with inhaled corticosteroids on the neutrophil and monocyte functions of asthmatic children. Our data showed that 6 months of ICS was not sufficient to return the decreased phagocyte functions to normal in the asthmatic children. Although 6 months of treatment caused some improvement in the clinical parameters, such as an increase in peak expiratory flow, and a significant increase in percentage of children with mild and moderate persistent asthma showing ACT > 25 and ACT = 20–24, (Table 2), no concomitant recovery of phagocyte function was observed.

Our data showed that the phagocytic capacity of monocytes and neutrophils in children with mild, moderate and severe persistent asthma was lower than in the healthy control children. A significant decrease in phagocytosis by monocytes and neutrophils [19,20], bronchial macrophages [21] and alveolar macrophages [11] has also been shown in asthmatic individuals.

Our data demonstrated that the severity of asthma did not influence the immunodeficiency in phagocytes. The phagocytic indices of the neutrophils and monocytes from the children with mild, moderate or severe persistent asthma were decreased, and there were no differences among these groups (Figure 1). Our data differed from those of Fitzpatrick et al. [11], who observed greater deficiencies in severe asthma patients. However, Alexis et al. [21] also showed decreased phagocytosis in mild asthma patients, whereas Lay et al. [22] observed increased phagocytosis by macrophages obtained from the sputum of asthma patients. The differences between our observations and those of other researchers who have assessed phagocytosis in asthma are most likely the result of the following factors: differences in the clinical form of the disease, the severity of the disease, the age of individuals, the treatment used, the stimuli used, the source of phagocytes tested, the cell type assessed, and genetic differences between individuals [11,19-22].

The deficiency in phagocytosis was evident when phagocytosis was assessed either through pathogen associated molecular pattern receptors (non-sensitized) or through complement and antibody receptors (sensitized). To be able to phagocytose, the phagocyte needs to move toward the *S. cerevisiae* and ingest the particle. Because the reduction in phagocytosis was attributable to the decreased quantitative involvement of the phagocytes in phagocytosis, it is possible that phagocytes in asthmatic children had decreased capacity to move toward the yeast. In fact, it was previously shown that β2-agonists and glucocorticoids, which are commonly used for the treatment of obstructive lung diseases, influence chemokine release and receptor sensitivity and, consequently, the chemotaxis of these cells [23].

When sensitized phagocytosis through FcγR and complement receptors was assessed, the deficiency was evidenced as both to the decreased quantitative involvement of the phagocytes in phagocytosis and the decreased number of yeast cells phagocytosed. The reasons for this effect are not clear; however, one possible explanation is a decrease in the expression of receptors for complement and/or FcγR in the membranes of the monocytes and neutrophils from asthma patients. Alexis et al. [19] found a significant correlation between the expression of CD64 (FcγR) and phagocytosis by bronchial macrophages from asthmatic individuals and the reduced expression of CD11b, a component of the CR3 receptor, and phagocytosis in sputum and in neutrophils and monocytes from asthmatics.

Although there was clinical improvement in asthma with corticosteroid therapy, we can hypothesize that the effect of the drug on phagocytes decreasing the phagocytosis may have contributed to the insufficient recovery of phagocytosis after six months of clinical follow-up. In fact, corticosteroids decrease phagocytosis by monocytes and the production of inflammatory cytokines [3,13]. Corticosteroids also increase the production of IL-10, a cytokine that deactivates monocytes [3,5]. Therefore, it is possible that phagocytosis did not return to normal because of the action exerted by the corticosteroids on the phagocytes. Another possibility to explain this lack of response of phagocytes to ICT by us observed might be that the insufficient recovery of immune alterations remained downmodulating phagocyte function.

Superoxide anion production, which was assessed as the per cent reduction of nitro blue tetrazolium, by phagocytes from asthmatic and control children was statistically decreased only for children with mild persistent asthma. The response to ICS after 6 months of treatment was different when assessed at baseline or after stimulation. Inhaled corticosteroids increased the per cent NBT reduction only when assessed without stimulation; however, these values were to the values of healthy, normal children, they did not exceed the values observed in normal children. However, when superoxide anion production was evaluated after stimulation with sensitized *S. cerevisiae*, there was a decrease in superoxide anion production. It is possible that decreased phagocytosis may have played a role in the decreased superoxide anion production by these cells.

Generation of radical oxygen species occurs through several enzymatic pathways or chemical process that are essential in many physiological reactions such as killing invading pathogens, and takes place in every cell. However, increased levels of ROS can produce harmful pathophysiological disorders that damage DNA, lipids, proteins, and carbohydrates, leading to enhanced inflammatory response [24]. Oxidative stress has been proven to affect smooth muscle contraction, induce airway hyper-responsiveness, and increase mucus secretion, and excessive ROS production can trigger key alterations, leading to an antioxidant-oxidant imbalance that has been shown in patients with asthma and differ significantly according to severity of the disease [24-26]. It was also observed that resting or stimulated phagocytes may produce differently reactive oxygen species that depends on the disease form [27]. Predominance of enhancement of ROS has been observed in asthma [25-27]. The differences between our observations and other researchers who have assessed ROS in asthma are most likely the result of the type of ROS evaluated, the cell type assessed and differences in the clinical form and severity of the disease.

Influence of treatment with corticoid has been also evaluated. It was observed that budesonide reduced the oxidative stress in the in guinea pig [28] and ICT was able to influence the production of NADP oxidase in a dose dependent manner [29]. Treatment with glucocorticosteroids as antioxidants has been suggested, based in its antioxidants properties [30]. We only observed influence of ICT in mild persistent asthma. A possible explanation by the different response observed by us is the type of ROS analyzed and the cell tested.

Oxidative injury leads to increased lipid peroxidation, increased airway reactivity and secretions, production of chemoattractant molecules, and increased vascular permeability [31-33], which collectively lead to the augmentation of the existing inflammation that is a hallmark of asthma. Therefore, lower oxygen radical production in asthma may contribute to the decreased immunopathogenesis by these molecules in asthma. On the other hand, a lower production of superoxide anions can lead to immunodeficiency and may hinder the immune defense against infectious agents.

Although corticosteroids are considered the first-line drugs for asthma therapy, the number of children that meet the clinical control evaluated by ACT (>25) in children that were followed-up for 6 m in this study was small. Significant variability in the response to inhaled corticosteroids for persistent asthma has been shown [34], and uncontrolled asthma occurs in more than 50% of children who receive treatment with low-dose inhaled corticosteroids [35]. It is possible that this lower clinical response occurred because there was a high percentage of moderate and severe persistent asthma among the assessed children. Furthermore, the children were treated at home, and although adherence to treatment was encouraged in all clinical evaluation, there is no assurance regarding the regular use of corticosteroids during the follow-up period. This uncertainty is a limitation of our study. The lower age of asthmatic children than healthy control children was also a limitation, however, we had already showed that there are not differences in phagocytosis between children higher 2 years old [16]. Some children received different ICS (budesonide or fluticasone) that is a limitation of this study. Another possible limitation of our study is the fact that phagocytosis was assessed in phagocytes obtained from peripheral blood and not from induced-sputum or bronchoalveolar lavage in order to evaluate directly the effects of disease and treatment on the phagocytic function at the local level. However, asthma is a systemic disease showing several cytokines and other molecules enhanced in peripheral blood that may influence functions of cells of immune system and phagocytosis and might be influenced by corticoid treatment as we observed in this paper. In support to this consideration, morphological changes in eosinophils obtained from peripheral blood markedly correlate with the disease and may indicate the clinical severity of the acute exacerbation [36], corroborating that blood cells may also be influenced by the immune response occurring in asthmatic individuals. Furthermore, sputum induction can be challenging for young children [37], and the influence on phagocytosis of mucus [38], enzymes [39] and microorganisms [40] presents in sputum couldn’t be excluded, in addition to the ethical limitation to evaluate phagocytosis in phagocytes from bronchoalveolar lavage.

## Conclusions

Our findings may broaden the understanding of the influence of inhaled corticosteroids on phagocyte functions. Our data showed that asthmatic children followed up for 6 months in treatment with ICT didn’t modify decreased phagocytosis. However, the baseline production of superoxide anions by phagocytes was normalized. Because phagocytes actively participate in both the lesion and the defense of the lung in asthma, it is possible that the result of these opposing effects was beneficial to treated patients because there was some clinical improvement. Furthermore, the reduction in lesions caused by oxygen radicals may have been beneficial to the treated children.

## Abbreviations

ICT: Inhaled corticosteroid therapy; ACT: Asthma control test; LABA: Long-acting β_2_-agonist; PBS: Phosphate-buffered saline; FCS: Fetal calf serum; NBT: Nitro blue tetrazolium; BMI: Body mass index; mPA: Mild persistent asthma; MPA: Moderate persistent asthma; SPA: Severe persistent asthma.

## Competing interests

The authors report no conflict of interests. The authors alone are responsible for the content of the paper. The authors used a copy-editing service for language revision.

## Authors’ contributions

CLFS-M and MIMJ designed the study protocol. CLFS-M and SCC performed the experiments. CLFS-M performed the clinical assessment. CLFS-M and MIMJ analyzed and interpreted the data. CLFS-M, SCC and MIMJ wrote the manuscript. MIMJ revised the manuscript. All authors read, revised and approved the final manuscript.
